# A novel report of a primary Merkel cell carcinoma lesion in the nasal vestibule

**DOI:** 10.1002/ccr3.1757

**Published:** 2018-09-12

**Authors:** Emily M. Khatchaturian, Narineh Zohrabian

**Keywords:** CK20, cutaneous malignancy, Merkel cell, skin cancer

## Abstract

Merkel cell carcinoma (MCC) accounts for less than 1% of cutaneous malignancies. As the lesion may mimic benign entities, clinicians’ differential should include rare entities to improve outcomes by early intervention. We present a case of primary MCC in the nasal vestibule requiring partial rhinectomy, suprahyoid lymphadenectomy, and radiation therapy.

## 1 BACKGROUND

Merkel cell carcinoma (MCC) is a rare, aggressive cutaneous malignancy that most frequently occurs in the head and neck region of the elderly population. Approximately 1,500 new cases will be diagnosed in the United States each year.^1^ Although the precise origin of the malignant cells remains contested, they share some resemblance to what Friedrich Sigmund Merkel initially described as “touch cells.” More recent studies illustrate that Merkel cells originate from neural crest.^2^ As such, the tumor may be more accurately described as cutaneous neuroendocrine carcinoma.^3^ However, clinicians continue to prefer the eponym Merkel cell carcinoma. In this study, we report a case of a primary MCC in the left nasal vestibule. The literature currently supports instances of primary MCC on the nasal septum and nasopharynx.^4^ We report a case of localized MCC in the nasal vestibule. This location has not frequently been cited in the literature.^5^

## 2 CASE REPORT

**Figure 1:**
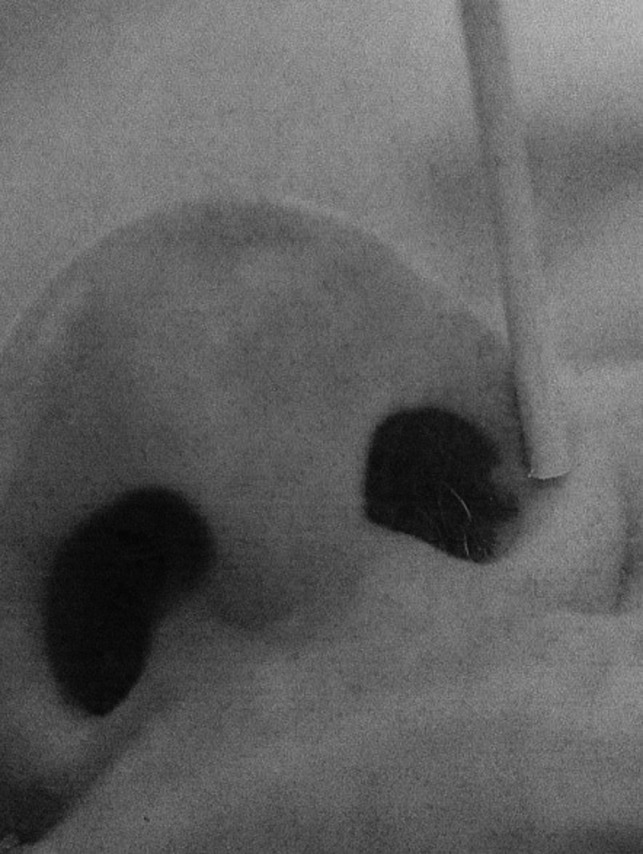
Papule within the left nasal vestibule

**Figure 2:**
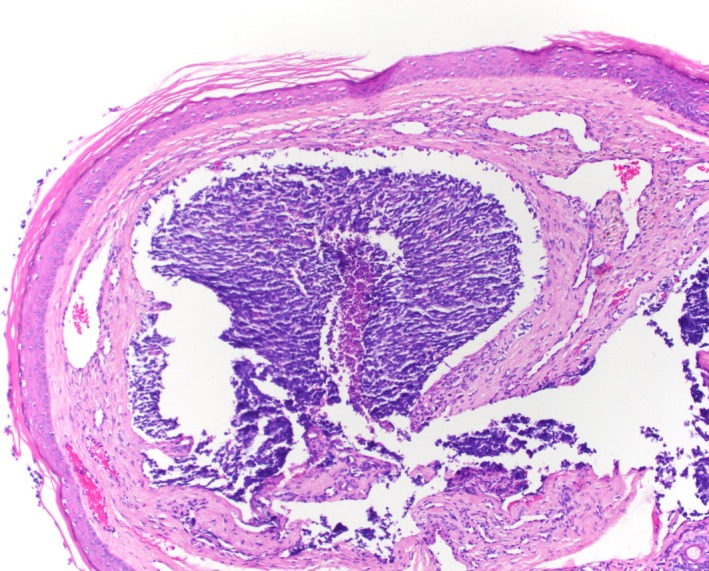
H&E, original magnification ×10

A 71‐year‐old white female with no previous history of skin cancer presented to the clinic for evaluation of lesions on her face in addition to her annual skin check. Family history for skin cancer was unknown as the patient was adopted. A 3‐mm translucent papule was noted on the left nasal ala and shaved for clinicopathologic evaluation to rule out cyst versus basal cell carcinoma (BCC) (Figure 1). Additionally, a 4‐mm pink papule on the right side of her nose was also shaved for histologic evaluation to rule out BCC. The 5 mm × 1 mm shave of the right side of nose returned as clusters of basaloid cells in the dermis with palisading nuclei and retraction spaces. A diagnosis of BCC was subsequently given and a Mohs resection was scheduled. The 3 mm × 1 mm shave biopsy of the left nostril showed expression of epithelial membrane antigen (EMA) and pankeratin, but not leukocyte common antigen (LCA). The laboratory reported a neoplasm of epithelial structure origin and recommended a second opinion. The pathology report for the second opinion noted cells of interest staining against AE‐1/AE‐3 in a perinuclear dot‐like pattern. EMA was also focally positive in a perinuclear pattern, and LCA was negative. The pathologist noted features suspicious for MCC (Figure 2) and recommended additional staining as well as complete re‐excision. Further studies showed the sample stained positive for chromagranin and neurofilament and negative for thyroid transcription factor (TTF‐1) Thus, the overall constellation of morphologic and immunohistochemical findings were compatible with a diagnosis of MCC. The patient was referred to the otolaryngologist who agreed with pursuing excisional biopsy of the left vestibular lesion.

## 3 MANAGEMENT AND OUTCOME

Sharp dissection was used to excise the lesion for excisional biopsy by the otolaryngologist. The tissue was submitted for histopathologic interpretation. A full‐thickness skin graft was then harvested from the postauricular region. The resection ultimately measured approximately 1.3 mm × 8 mm and was located just inside the left alar rim. Grossly, the specimen exhibited a slightly erythematous, firm lesion with some radial telangiectasias. No gross invasion into the underlying alar cartilage was noted.

**Figure 3:**
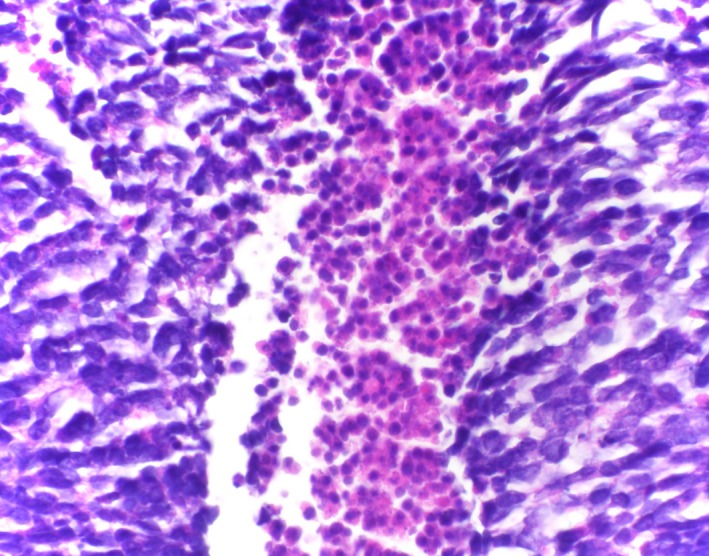
H&E, original magnification ×60 objective with necrosis

**Figure 4:**
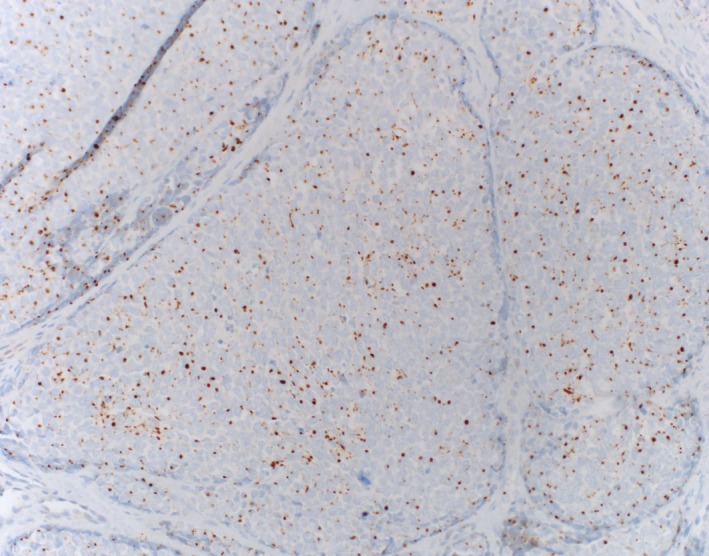
Dot like CK20, original magnification ×20

**Figure 5:**
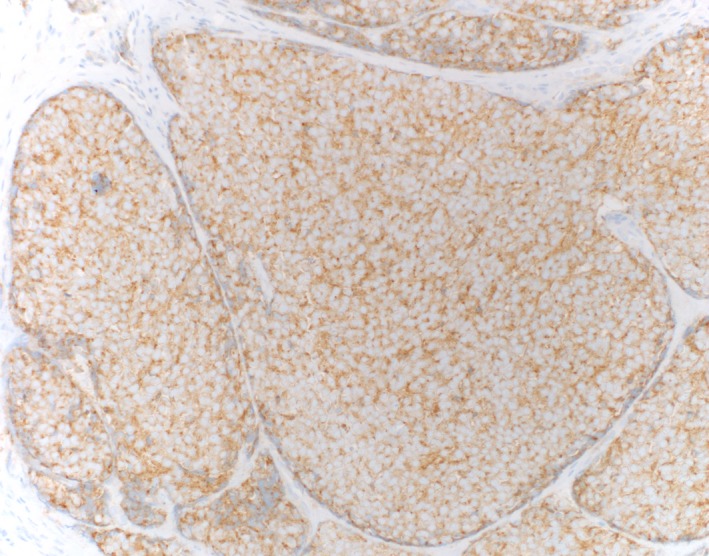
Synaptophysin, original magnification ×20

The surgical pathology report indicated a 2.5 mm focus of invasive carcinoma in the center of the specimen with the tumor infiltrating in a nested pattern. The tumor cells showed extremely high N:C ratio with nuclear molding, fine chromatin, small nucleoli, brisk mitotic activity, and abundant individual cell necrosis (Figure 3). These morphologic findings raised a differential diagnosis between MCC and metastatic small cell carcinoma of pulmonary origin. A panel of immunohistochemical stains demonstrated positive staining for CAM5.2 and CK20 in a characteristic dot‐like pattern (Figure 4). They also expressed neuron‐specific enolase (NSE), synaptophysin, CD56, and neurofilament protein (Figure 5). The cells of interest were negative for CK7 and TTF‐1. This immunoprofile is consistent with a diagnosis of Merkel cell carcinoma and argues against a metastatic small cell carcinoma.

The patient was scheduled for partial rhinectomy and left suprahyoid lymphadenectomy**.** On final pathology, the diameter of the malignancy was 2.5 mm. There had been a previous specimen measuring 3.1 mm. In total size, the specimen was estimated at approximately 6 mm. Reconstruction was performed of the left nasolabial flap. Left suprahyoid neck dissection included levels 1A, 1B, and 2A with preservation of the submandibular gland.

On one‐month follow‐up, we discussed radiation therapy adjuvant and PET scan with the patient. A total body skin exam (TBSE) showed no evidence of recurrence or lymphadenopathy. Two months after surgery, a PET scan from the skull base to midthighs showed no definite evidence of residual nasal or metastatic disease. No lymphadenopathy, evidence of recurrence, or new suspicious lesions were noted at the two‐month follow‐up. Additionally, laboratory tests for CBC with auto differential, CMP, and LDH were performed and values returned within normal ranges. By the five‐month follow‐up, the patient had completed radiation therapy to the area and returned to the clinic for TBSE. On exam, there remained no evidence of recurrence. Continued follow‐up through fourteen months showed no evidence of recurrence or metastasis by PET scan, CT imaging and laboratory analysis.

## 4 DISCUSSION

MCC is a highly radiosensitive tumor that typically appears as a single, painless lump on sun‐exposed skin. The differential diagnosis includes basal cell carcinoma, cutaneous melanoma, dermatofibroma, and keratoacanthoma (Table 1).^6^ The malignant cells tend to spread locally via the lymphatics system. Researchers have suggested that this tumor spreads by an orderly cascade pattern in which elective regional lymph node dissection is reasonable.^7^

**Table 1:** Differential diagnosis of Merkel cell carcinoma

Basal cell carcinoma
Cutaneous melanoma
Dermatofibroma
Keratoacanthoma

Chronic sun exposure and an immunocompromised state may affect the risk of MCC. This link to immunosuppression led to the discovery of Merkel cell polyomavirus in 2008 by Feng et al.^8^ Additional risk factors for the development of MCC also include age greater than 50, male gender, or white ethnicity. Factors predictive of improved survival include head and neck tumor site and negative lymph nodes at presentation.^7^ Of note, tumor size has had no impact on survival.^9^

Histologically, MCC resembles metastatic small cell carcinoma of pulmonary origin. It may also resemble melanoma which is often referred to as the “great mimicker” with its wide spectrum of possible histologic features.^10^, ^11^ Therefore, immunohistochemical staining is often used to make the diagnosis of MCC. CK20 is a highly specific marker for MCC. Indeed, less than 5% of MCC specimens lack CK20 expression.^12^ Additionally, neuron‐specific enolase and neurofilament protein are sensitive for MCC (Table 2).^13^ In contrast, TTF‐1 is positive in metastatic small cell lung carcinoma (SCLC) but negative in MCC.^14^ LCA (CD45) and cytokeratin 7 (CK7) are positive in lymphoma and SCLC, respectively, but negative in MCC.^15^, ^16^, ^17^ Melanoma is S100 positive.

**Table 2:** Immunohistochemistry of Merkel cell carcinoma^13^

	CK20	CK7	NSE	TTF‐1	S100	LCA
Merkel cell carcinoma (MCC)	+	−	+	−	−	−
Small cell carcinoma of the lung (SCLC)	−	+	+	+	−	−
Melanoma	−	−	−	−	+	−
Lymphoma	−	−	−	−	−	+

CK20: cytokeratin 20; CK7: cytokeratin 7; LCA: leukocyte common antigen; NSE: neuron‐specific enolase; TTF‐1: thyroid transcription factor 1.

Radical surgical excision with pathologic verification of complete removal of the tumor is the recommended treatment. Early MCC can be effectively treated by surgery with or without postoperative radiation therapy, whereas advanced MCC is currently considered incurable.^18^

## 5 CONCLUSION

Our patient is unique because the lesion was found in an atypical location of the nasal vestibule. Merkel cell carcinoma is a rare tumor, currently accounting for less than 1% of cutaneous malignancies.^6^ The 5 year disease‐specific survival rate is 64%. However, patients diagnosed with localized MCC have a 5‐year relative survival of 75%.^18^ As differential diagnosis includes benign entities such as dermatofibroma, clinicians should keep more rare diagnosis in their differential, as well, in order to warrant early intervention and improved patient outcomes. At the time of submission of this publication, the patient continues to do well greater than four years out from her initial diagnosis.

## CONFLICT OF INTEREST

None declared.

## AUTHORSHIP

NZ: conceived the idea. EK: wrote the manuscript including revisions with support from NZ. The final manuscript was evaluated and approved by both authors.
